# Coinfección por SARS-CoV-2 y rinovirus-enterovirus en una paciente adulta joven críticamente enferma en Colombia

**DOI:** 10.7705/biomedica.5516

**Published:** 2020-11-12

**Authors:** Juan Pablo Orozco-Hernández, Juan José Montoya-Martínez, Manuel Conrado Pacheco-Gallego, Mauricio Céspedes-Roncancio, Gloria Liliana Porras-Hurtado

**Affiliations:** 1 Grupo de Investigación Salud Comfamiliar, Clínica Comfamiliar, Pereira, Risaralda, Colombia Grupo de Investigación Salud Comfamiliar PereiraRisaralda Colombia; 2 Programa de Medicina, Universidad Tecnológica de Pereira, Risaralda, Colombia Universidad Tecnológica de Pereira Universidad Tecnológica de Pereira Risaralda Colombia; 3 División de Neumología y Endoscopia Respiratoria, Departamento de Medicina Interna, Clínica Comfamiliar, Pereira, Risaralda, Colombia Departamento de Medicina Interna Clínica Comfamiliar PereiraRisaralda Colombia

**Keywords:** infecciones por coronavirus, neumonía, síndrome de dificultad respiratoria del adulto, informes de casos, Rhinovirus, Colombia, Coronavirus infections, pneumonia, respiratory distress syndrome, adult, case reports, Rhinovirus, Colombia

## Abstract

La actual pandemia por SARS-CoV-2 ha ocasionado un enorme problema de salud pública mundial. Se reporta el caso de una paciente adulta joven con SARS-CoV-2 confirmado por laboratorio. Se describe la identificación del virus y el curso clínico, el diagnóstico y el tratamiento de la infección. La paciente tuvo un rápido deterioro clínico a partir de síntomas iniciales leves que progresaron a una neumonía multilobar que requirió su hospitalización en la unidad de cuidados intensivos.

Se destaca la importancia de establecer un diagnóstico basado en la clínica y los antecedentes del paciente, y considerando los posibles síntomas gastrointestinales además de los respiratorios. Asimismo, debe indagarse sobre la presencia de factores de riesgo, en este caso, la obesidad. También se señalan las limitaciones en las pruebas diagnósticas y la posibilidad de infección concomitante con otros agentes patógenos respiratorios, así como los hallazgos en las imágenes diagnósticas, los exámenes de laboratorio y el tratamiento en el marco de la limitada información con que se cuenta actualmente.

## Abstract (en)

The current SARS-CoV-2 pandemic has caused a huge global public health problem. We report the case of a young adult patient with laboratory-confirmed SARS-CoV-2. We describe the identification of the virus and the clinical course, diagnosis, and treatment of the infection including her rapid clinical deterioration from the mild initial symptoms, which progressed to multilobar pneumonia requiring admission to the intensive care unit. This case highlights the importance of establishing a diagnosis based on the clinical findings and the patient's history bearing in mind the possibility of gastrointestinal symptoms in addition to respiratory ones. Besides, the presence of risk factors should be investigated; in this case, we proposed obesity as a possible risk factor. Furthermore, limitations in diagnostic tests and the possibility of co-infection with other respiratory pathogens are highlighted. We describe the imaging, laboratory findings, and treatment taking into account the limited current evidence.

La actual pandemia por el nuevo coronavirus (SARS-CoV-2) ha sido un enorme reto y un problema para la salud pública mundial desde su origen en la ciudad de Wuhan, China ^1^. Hasta el 23 de abril de 2020, la infección por SARS-CoV-2 había infectado alrededor de 2'588.068 de individuos y ocasionado 182.808 muertes a nivel global, con 4.561 infectados y 215 muertes en Colombia ^2^. Se han publicado estudios observacionales que indican que en 46 a 84 % de los pacientes el curso clínico es leve, en 15 a 25 %, grave y en el 5 %, crítico, en tanto que el 2,3 % requieren respiración mecánica invasiva ^1^^,^^3^^,^^4^. La letalidad ha sido variable entre países, con un promedio entre 2 y 3 %; sin embargo, debido al subregistro y al elevado número de pacientes asintomáticos se informan tasas de menos del 1 % ^5^.

En el diagnóstico del SARS-CoV-2 se han descrito una serie de hallazgos clínicos, de laboratorio y de imágenes diagnósticas que incluyen la presencia de fiebre (89 %), tos (67 %), fatiga (38 %), diarrea (3,8 %), mialgias y artralgias (15 %) y disnea (19 %) ^1^. Asimismo, se han caracterizado factores de predicción de la gravedad y la mortalidad como la edad avanzada, las comorbilidades, la disnea, la taquipnea, y la elevación en los valores de la troponina, la ferritina, la lactato deshidrogenasa (LDH) y el dímero D ^4^.

En el contexto del desconocimiento actual de varios aspectos del curso clínico de la infección por SARS-CoV-2, teniendo presente que los reportes de caso han aportado valiosa información clínica y científica, y del hecho de que los factores de riesgo pueden diferir entre los países de altos ingresos y aquellos de bajos y medianos ingresos, se describe el caso de una paciente adulta joven que presentó un cuadro crítico por infección concomitante de SARS-CoV-2 y rinovirus-enterovirus, con obesidad, neumonía multilobar y resultado negativo inicial en la reacción en cadena de la polimerasa en tiempo real (PCR-RT), que posteriormente dio positivo para SARS-CoV-2.

Además, se destaca la importancia de una aproximación basada en la clínica, los antecedentes y los factores de riesgo del paciente, teniendo presente la limitación de las pruebas diagnósticas, la posibilidad de síntomas gastrointestinales y la infección con otros agentes patógenos respiratorios. Asimismo, se describen los hallazgos en las imágenes diagnósticas, los exámenes de laboratorio y el tratamiento aplicado a partir de la escasa evidencia actual.

## Reporte de caso

El 12 de marzo de 2020, una paciente de 41 años consultó al servicio de urgencias de una clínica de Cartago (Colombia) con un cuadro clínico de seis días de tos seca, congestión nasal, fiebre, odinofagia, fatiga, náuseas, diarrea e hiporexia. No presentaba anosmia, ageusia u otros síntomas. La paciente había tomado azitromicina automedicada durante tres días (500 mg por día) sin resultados. Refirió que había llegado de Nueva York (Estados Unidos) el 10 de marzo con su hermano, quien había tenido diagnóstico confirmado de SARS-CoV-2; el único antecedente médico relevante de la paciente era la obesidad. Se le tomó una muestra con hisopado nasofaríngeo en el día 6 de la enfermedad, la cual fue negativa para SARS-CoV-2, por lo que se ordenó su egreso con indicaciones de aislamiento domiciliario, acetaminofén y loratadina.

La paciente presentó un deterioro clínico con disnea, lo que la llevó a consultar de nuevo el 14 de marzo (día 8 de la enfermedad) y fue hospitalizada; se le hicieron estudios de laboratorio y se le tomó una radiografía inicial que evidenció la presencia de opacidades multilobares con patrón de vidrio esmerilado. Se inició el tratamiento con 500 mg de cloroquina, 400 mg de lopinavir y 100 mg de ritonavir cada 24 horas por vía oral.

**Figura 1:**
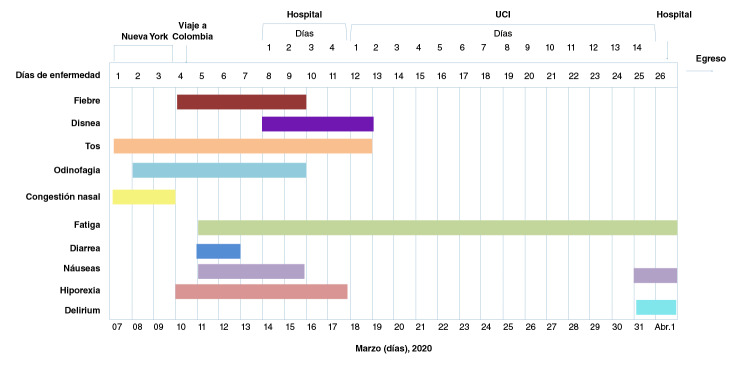
Síntomas de la paciente en función del tiempo (7 de marzo al 1^o^ de abril)

El 17 de marzo (día 11 de la enfermedad) se tomó una segunda muestra de hisopado nasofaríngeo, pues había una clara sospecha clínica de infección por el nuevo coronavirus. La paciente permaneció hospitalizada en Cartago hasta el 18 de marzo, pero dada el agravamiento de su cuadro clínico respiratorio (necesidad de oxigenoterapia de alto flujo), fue remitida a una institución de mayor complejidad (figura 1).

En el momento de ingreso a la clínica en Pereira la paciente pesaba 84 kg, la talla era de 1,60 m, su índice de masa corporal era de 32,8 (kg/m^2^), y tenía tensión arterial de 102/65 mm Hg, frecuencia respiratoria de 26 respiraciones por minuto, frecuencia cardiaca de 110 latidos por minuto, saturación de oxígeno (Sa0_2_) al 96 % con 3 L/min por cánula nasal y temperatura axilar de 36,4 °C. Su condición era aceptable, estaba consciente y orientada, pero con taquipnea y polipnea.

Los resultados de laboratorio de su primera hospitalización en Cartago indicaban un aumento de la LDH, la proteína C reactiva, leucocitosis elevada con neutrofilia y un panel respiratorio viral negativo para todos los virus evaluados, incluidos los de influenza A y B, parainfluenza, virus respiratorio sincitial, rinovirus, adenovirus y los cuatro coronavirus comunes en el medio (HKU1, NL63, 229Ey OC43) (cuadro 1).

**Cuadro 1:** Resultados de los exámenes de laboratorio clínico

Prueba (unidad de medida)	Rango de referencia	Días 7 a 10 de la enfermedad, días 1-5 de hospitalización	Día 12 de la enfermedad, día 1 en UCI	Día 13 de la enfermedad, día 2 en UCI	Día de la enfermedad 18 y día 7 en UCI	Día de la enfermedad 25 y 14 en UCI
Recuento de leucocitos (por µl)	4.000-10.000	20.810	8.700	10.510	15.500	8.870
Recuento de neutrófilos (por µl)	1.820-7.420	18.104	6.480	8.220	12.600	6.400
Recuento de linfocitos (por µl)	850-3.740	1.664	1.260	1.200	1.170	1.410
Hemoglobina (mg/dl)	11,2-15,7	"adecuado"	14,1	13,6	13,1	11
Hematocrito (%)	34-45	"adecuado"	43	40	39	32
Plaquetas (por µl)	151.000-400.000	"adecuado"	340.000	373.000	277.000	393.000
Proteína C reactiva (mg/L)	0-5	24	74	-	-	12
Lactato deshidrogenasa (U/L)	0-223	230	-	406	522	-
Sodio (mmol/L)	135-149	135	138	137	136	140
Potasio (mmol/L)	3,5-5,1	4.0	4,2	4,2	4,5	3,3
Cloro (mmol/L)	98-107	98	102	98	96	-
Calcio (mmol/L)	8,4-10,2	"adecuado"	9,0	-	-	-
Glucosa (mg/dl)	60-100	106	94	104	110	88
Creatinina (mg/dl)	0,4-0,95	0,67	0,56	0,48	-	0,75
Nitrógeno ureico en sangre (mg/dl)	6-20	6,6	8,1	6,5	-	-
P02 (mm Hg)	80-100	66	108	101	69	119
PC02 (mm Hg)	35-45	35	50	45	61	32
HC03 (mm Hg)	-	24	24	25	34	21
pH arterial	7,35-7,45	7,46	7,3	7,37	7,35	7,43
Bilirrubina total (mg/dl)	0-1	-	-	0.5	-	-
Alanina transaminasa (U/L)	0-31	-	-	43	176	85
Aspartate transaminasa (U/L)	0-31	-	-	43	247	85
Lactato arterial (mmol/L)	0,5-2,2	-	-	1,46	-	-
Tiempo de trombina (s)	9,9-11,8	-	-	9,9	10,8	-
Tiempo de tromboplastina (s)	25-31,3	-	-	26,9	32,5	-
Razón internacional normalizada	-	-	-	0,9	1,0	-

Con base en estos hallazgos y con la sospecha de una neumonía grave de origen viral, se le hicieron nuevos estudios de laboratorio y se inició la administración de 600 mg de n-acetilcisteína cada 8 horas, 4,5 g de piperacilina-tazobactam cada 6 horas, aumento de las dosis de cloroquina, lopinavir y ritonavir a 500, 400 y 100 mg, respectivamente, cada 12 horas por vía oral (día 11 de la enfermedad).

**Figura 2:**
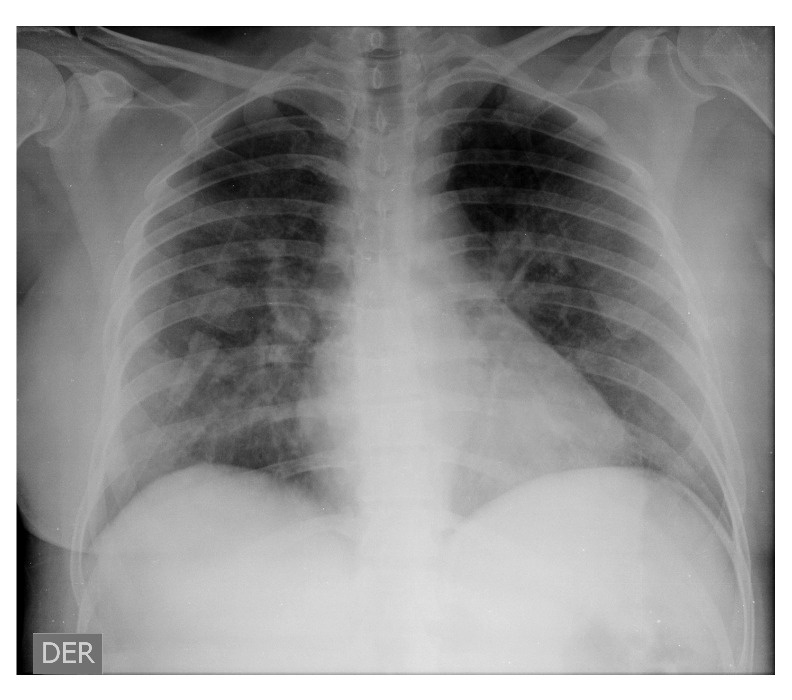
Radiografia postero-anterior de torax, 18 de marzo de 2020 (dia 12 de la enfermedad). Se observan opacidades con patron de vidrio esmerilado.

El mismo día de su ingreso, la paciente tuvo un rápido deterioro respiratorio y presentó un trastorno grave de la oxigenación, con PaFi de 99 (PaO_2_ de 49,7 mm Hg y FiO_2_ de 50 %); en la radiografía de tórax se observaron opacidades con patrón de vidrio esmerilado, por lo que se hizo la intubación endotraqueal y se la trasladó a la unidad de cuidados intensivos donde se le suministraron líquidos endovenosos (60 ml/hora de lactato de Ringer), protección gastrointestinal, tromboprofilaxis y respiración mecánica invasiva en posición de pronación en modo asistido controlado con elevada presión positiva al final de la espiración (PEEP) de 10 cm de H_2_O y volumen corriente de 6 ml/kg (figura 2).

**Figura 3:**
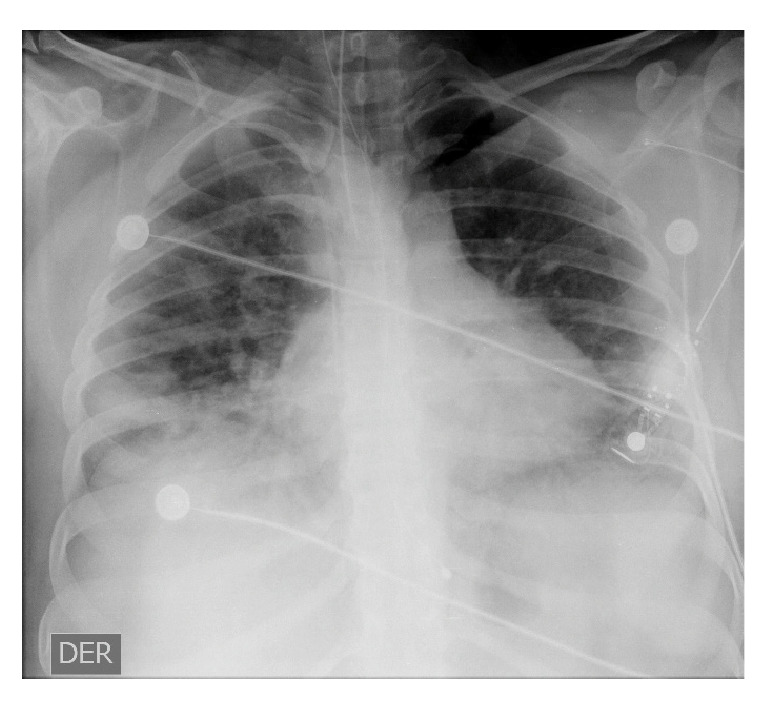
Radiografia antero-posterior de torax, 19 de marzo de 2020 (dia 13 de la enfermedad). Se observan opacidades multifocales bilaterales de predominio periferico (sugestivas de neumonia multilobar).

En los días 1 a 6 en la unidad de cuidados intensivos, la paciente se encontraba estable, con permanente y estricto aislamiento por contacto y por gotas; no presentaba dificultad respiratoria ni fiebre, pero sí taquicardia; el hemograma no registraba alteraciones y hubo mejoría en la gasometría, con un leve trastorno de la oxigenación, y radiografías con evidencia de opacidades multifocales (figuras 3 y 4).

**Figura 4:**
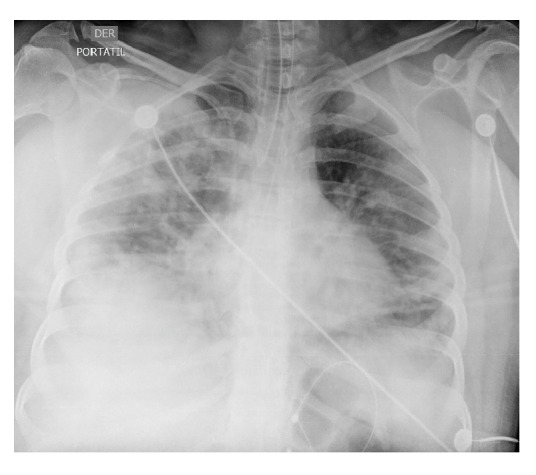
Radiografia antero-posterior de torax, 23 de marzo de 2020 (dia 17 de la enfermedad). Se observan opacidades reticulo-alveolares de predominio periferico y derecho.

El día 20 de marzo (día 14 de la enfermedad y 3 en la unidad de cuidados intensivos) se recibió el resultado positivo para SARS-CoV-2. La paciente se mantuvo en relajación neuromuscular con cisatracurio hasta el día 5 de su estancia en la unidad de cuidados intensivos, cuando esta fue suspendida, pero el trastorno respiratorio empeoró, con disminución de la PaFi (218 a 152) en un día, por lo que en el día 6 (día 17 de la enfermedad) se reinició este tratamiento y la paciente tuvo mejoría clínica.

**Figura 5:**
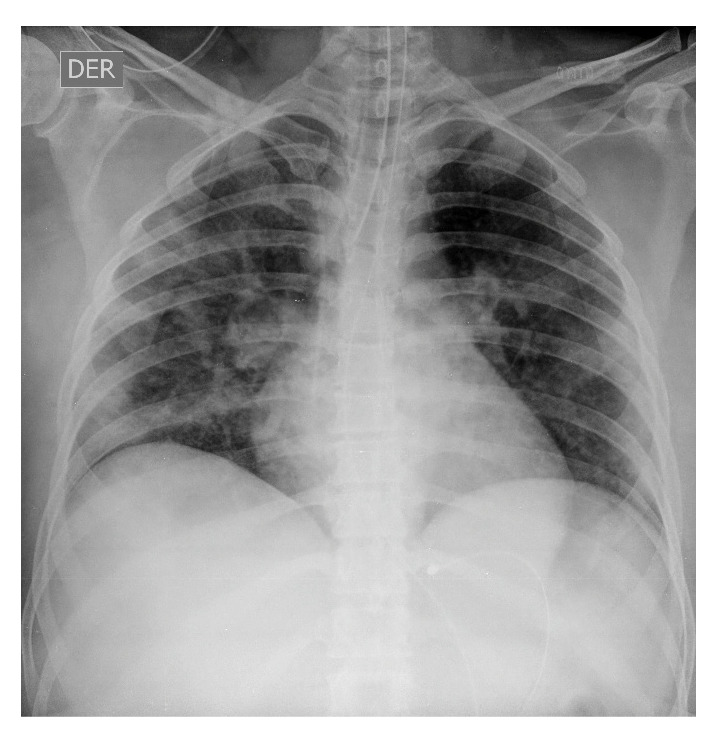
Radiografía antero-posterior de tórax, 24 de marzo de 2020 (día 18 de la enfermedad). Se observa mejoría con respecto a las imágenes previas.

Se tomó una nueva radiografía de control (figura 5) en la que se observó mejoría radiológica. En el día 7 en la unidad de cuidados intensivos, la paciente continuaba en el mismo estado: elevación de transaminasas, tiempos de coagulación y persistencia de LDH, hemograma con leucocitosis y gases con trastorno leve a moderado de oxigenación, leve acidosis respiratoria e hipercapnia (cuadro 1), por lo que se suspendió el lopinavir y el ritonavir, y se continuó con los demás tratamientos descritos, incluida la respiración mecánica invasiva con presión positiva al final de la espiración de 11 cm de H_2_O, volumen corriente de 6 ml/kg y posición en pronación con ciclos cortos de supinación.

Entre los días 8 y 13 la paciente presentó una significativa mejoría respiratoria (PaFi de 267) y de la gasometría, por lo que en el día 8 se suspendieron los antibióticos y los relajantes musculares.

En el día 14 de su estancia en la unidad de cuidados intensivos (día 24 de la enfermedad) se reportó en el panel viral el aislamiento de rinovirus y enterovirus humanos.

La condición clínica de la paciente siguió mejorando, despertaba sin dificultades y se retiró exitosamente la intubación endotraqueal, por lo que salió de la unidad de cuidados intensivos, permaneciendo otro día en el hospital, cuando se decidió darle el egreso ante su clara mejoría clínica.

El tiempo de estancia en la unidad de cuidados intensivos fue de 14 días. El 29 de marzo se le tomó un nuevo hisopado nasofaríngeo para la detección del coronavirus, el cual fue negativo. La paciente presentó un delirio hiperactivo que se resolvió, por lo que se ordenó su egreso con seguimiento ambulatorio durante el cual no tuvo nuevos síntomas.

### Consideraciones éticas

El reporte de caso fue aprobado por el Comité de Ética de la Clínica Comfamiliar y fue clasificado como un estudio "sin riesgo" La paciente firmó el consentimiento informado.

## Discusión

Este reporte de caso es un aporte importante al manejo de la infección por SARS-CoV-2 surgida en Wuhan (China), que luego se diseminó alrededor del mundo, incluido Colombia, convirtiéndose en pandemia ^1^^,^^3^^,^^6^. La paciente de este caso provenía de Estados Unidos, país que actualmente registra el mayor número de casos a nivel mundial ^7^, donde ella y su hermano se infectaron. Todavía se desconocen varios aspectos del curso clínico de esta enfermedad, principalmente en cuanto a complicaciones y probables secuelas a largo plazo.

Entre los aspectos analizados en este caso se encuentran los síntomas respiratorios iniciales de la paciente, descritos previamente en estudios observacionales ^1^^,^^3^^,^^4^^,^^6^, pero también otros poco descritos como los gastrointestinales (diarrea, hiporexia y náuseas).

Los síntomas gastrointestinales en la infección por SARS-CoV-2 inicialmente descritos en Wuhan tenían una baja prevalencia, de solo 3,8 %, probablemente debido a su subregistro por la mayor atención dada a los síntomas respiratorios ^1^. Tian, *et al.*^8^, revisaron los datos de 2.023 pacientes con infección por SARS-CoV-2 y evaluaron la presencia o ausencia de síntomas gastrointestinales. En su revisión establecieron que entre el 3 y el 79 % de los pacientes había presentado síntomas gastrointestinales, siendo la anorexia o hiporexia (39,9-50 %), la diarrea (2-49,5 %) y las náuseas (1-29,4 %) los más frecuentes, los que también presentó la paciente de este caso.

La paciente tampoco presentó anosmia y ageusia, síntomas descritos solo en algunos escasos reportes ^9^. Los síntomas respiratorios y gastrointestinales se deben a la invasión del virus en los neumocitos de tipo 2 y en los enterocitos de colon e íleon, lo que produce malabsorción. En estudios de bioinformática y transcriptómica se ha descrito que expresan el receptor de la enzima convertidora de angiotensina 2 (ECA2), lo cual se ha confirmado con los hallazgos en biopsias y muestras fecales de pacientes con el virus ^10^.

Es importante resaltar el rápido deterioro clínico de la paciente, cuyos síntomas iniciales fueron leves, pero posteriormente progresaron a una neumonía multilobar con síndrome de dificultad respiratoria del adulto similar a lo descrito en otros reportes ^1^^,^^11^.

En la evolución de los pacientes con formas graves de SARS-CoV-2 se ha descrito la presencia de un componente inflamatorio bautizado como "tormenta de citocinas" que incluye síntomas como fiebre constante, citopenias e hiperferritinemia, progresión al síndrome de dificultad respiratoria del adulto y un perfil de citocinas caracterizado por un aumento de interleucina (IL)-2, IL-7, del factor estimulante de colonias del granulocito, el interferón y el factor de necrosis tumoral-a, todos incluidos entre los factores de predicción de la mortalidad descritos en el estudio retrospectivo multicéntrico de 150 casos confirmados de SARS-CoV-2 en Wuhan, China, y asociados con la elevación de la ferritina y el IL-6 (p<0,001), lo que sugiere que la mortalidad puede deberse a una hiperinflamación promovida por el virus ^12^.

Este fenómeno inflamatorio generalmente ocurre alrededor del día 7, cuando los cuadros clínicos empeoran, lo que coincide con lo sucedido en el presente caso, pues fue en el día 8 que la paciente tuvo que volver a consultar y se le detectaron signos de alarma.

Asimismo, se debe hacer énfasis en los factores de predicción de la gravedad y la mortalidad hasta ahora descritos: la edad avanzada, las comorbilidades, la disnea, la taquipnea (definida como una frecuencia respiratoria mayor o igual a 24), la linfopenia, la elevación de los niveles de troponina, ferritina, lactato deshidrogenasa (LDH) y dímero D ^1^^,^^4^^,^^13^^,^^14^.

En cuanto a la paciente de este caso, se determinaron como factores de riesgo de gravedad, la edad y su antecedente de obesidad, aspecto que no ha sido analizado en los estudios de Italia o Wuhan ^1^^,^^3^^,^^4^ y que podría asociarse con la inflamación intrínseca en esta enfermedad ^15^. Además, la paciente presentó disnea, taquipnea, elevación de la LDH y la proteína C reactiva, que se han descrito como factores de predicción de la gravedad. Asimismo, se destaca la coagulopatia que presentó el día 18 de su enfermedad, hallazgo descrito previamente como una complicación ^1^.

Cabe mencionar la limitación de la PCR-RT para el diagnóstico de SARS-CoV-2, cuya sensibilidad se ha reportado como modesta, probablemente por su vulnerabilidad a las inadecuadas condiciones previas al análisis ^16^. En este caso la paciente tuvo una primera prueba negativa realizada el día 6 de la enfermedad, lo que pudo deberse, en parte, al día de evolución de la enfermedad, ya que la recomendación actual del Instituto Nacional de Salud es realizarla a partir del día 7 ^2^. En este sentido, en un estudio reciente se estableció que la PCR-RT puede ser positiva incluso después de dos pruebas negativas en el 21,4 % de los casos, lo que sugiere que estos falsos negativos podrían explicarse por un prolongado aclaramiento viral o por la conversión de ácidos nucleicos ^17^.

En cuanto a la infección concomitante de SARS-CoV-2 y rinovirus-enterovirus en esta paciente, en el estudio de Kim, *et al.*^18^, se evidenció que el 116 (9,5 %) de los 1.217 pacientes con sintomatología respiratoria dio positivo para SARS-CoV-2 y 318 (26,1 %) para otro microorganismo diferente. Del grupo positivo para SARS-CoV-2, 24 (20,7 %) también lo fue para uno o más agentes patógenos adicionales, siendo los más frecuentes los rinovirus y los enterovirus (n=8; 6,9 %), el virus sincitial respiratorio (n=6; 5,2 %) y otros coronavirus (n=5; 4,3 %) ^18^. En otro estudio de 115 pacientes con infección por SARS-CoV-2, 5 (4,35 %) también tenían infección por influenza ^19^. Khodamoradi, *et al.,* reportaron una serie de cuatro casos que cursaron con neumonía grave y presentaban infección concomitante por SARS-CoV-2 e influenza de tipo A ^20^.

Por lo tanto, se recomienda que el panel respiratorio de PCR múltiple (PR-FilmArray) se haga solo para pacientes graves y aquellos en quienes el resultado positivo obligue a modificar el tratamiento para prevenir la progresión de la enfermedad e, incluso, la muerte ^18^.

En la actualidad, hay varios tratamientos en fase de experimentación, pero la información confirmada todavía es poca para recomendarlos en las guías de práctica clínica, en tanto que el desarrollo de las vacunas se encuentra en diferentes etapas ^21^^,^^22^. El uso de la cloroquina y la hidroxicloroquina tiene el mayor respaldo hasta el momento como tratamiento benéfico para pacientes con SARS-CoV-2 ^23^, aunque debe tenerse presente que se recomiendan a partir de estudios con importantes sesgos de selección e información que han recurrido a resultados surrogados y tamaños de muestra reducidos; además, deben considerarse los posibles riesgos adversos, como la prolongación del intervalo QT y la muerte súbita.

También se ha evaluado la azitromicina administrada conjuntamente con hidroxicloroquina en un estudio no aleatorizado de 36 pacientes en Francia, utilizando un resultado surrogado (carga viral) y descartando otros de importancia clínica y para el paciente ^24^. En el estudio de Gautret, *et al.,* de diseño observacional no controlado, se evaluó la evolución de 80 pacientes con infección por SARS-CoV-2 leve, tratados con azitromicina e hidroxicloroquina, lo que disminuyó la carga viral y el tiempo de estancia en la unidad de infecciones ^25^. A pesar de la escasa evidencia, estos resultados son alentadores.

El uso de lopinavir y ritonavir en un reciente ensayo clínico no aleatorizado no demostró un beneficio evidente ^26^.

En este caso, se utilizaron varios medicamentos en estudio, incluidos antibióticos de amplio espectro, sin que pudiera determinarse la eficacia individual de cada uno en la mejoría clínica de la paciente puesto que se administraron simultáneamente. El único medicamento utilizado de forma individual y automedicado al inicio de la enfermedad fue la azitromicina, que no tuvo eficacia.

Se utilizaron las recomendaciones para la respiración mecánica invasiva actuales, como el uso de una elevada presión positiva al final de la espiración, la respiración en pronación por ciclos y un volumen corriente entre 4 y 8 ml/kg, lo que fue relevante en la mejoría clínica observada ^27^.

Por último, el delirio posterior al retiro de la intubación de la paciente pudo responder a diversos factores, pero se sugiere la posibilidad de una complicación del tipo de una encefalitis viral por SARS-CoV-2 ya descrita en la literatura especializada. Se ha evidenciado el neurotropismo y la neurovirulencia del virus, el cual tiene una vía de ingreso neuronal a través del epitelio y del bulbo olfatorio, y dada la alta expresión de la ECA2 en las neuronas y las células gliales ^28^.

En este caso se destacan varios aspectos relevantes para el diagnóstico y el manejo de los pacientes con infección por SARS-CoV-2:

1. el diagnóstico debe basarse en la sospecha clínica (historia, resultados de laboratorio e imágenes) y los antecedentes de viajes o contactos;
2. se recomienda indagar sobre los factores de riesgo descritos en la literatura, y se propone incluir la obesidad como uno de ellos;
3. se deben tener presentes los síntomas no respiratorios del SARS-CoV-2 para el diagnóstico, como son los gastrointestinales y los neurológicos;
4. se resaltan las limitaciones de las pruebas diagnósticas, por lo que una primera PCR-RT negativa no debe descartar el diagnóstico cuando la sospecha clínica lo indique, ya que puede haber falsos negativos;
5. debe evaluarse la posibilidad de infección concomitante con otros agentes patógenos respiratorios que pueden generar duda en el diagnóstico y se recomienda utilizar PR-FilmArray en casos graves o en aquellos en que el resultado positivo pueda modificar el tratamiento a favor del pronóstico del paciente;
6. debe mantenerse un estricto aislamiento por gotas y por contacto del paciente con sospecha o confirmación de SARS-CoV-2 utilizando siempre los elementos de protección personal y el lavado de manos, y
7. se debe tener presente la terapéutica y sus posibles efectos adversos dada la escasa evidencia actual, siendo la terapia respiratoria con posición en pronación ajustada a las recomendaciones del consenso nacional ^29^ y la guía internacional ^27^, un pilar fundamental en el tratamiento.
